# Rationing for medicines by health care providers in Indonesia National Health Insurance System at hospital setting: a qualitative study

**DOI:** 10.1186/s40545-019-0170-5

**Published:** 2019-05-07

**Authors:** Endang Yuniarti, Yayi Suryo Prabandari, Erna Kristin, Sri Suryawati

**Affiliations:** 1 Pharmacy Program, Muhammadiyah Health Science Institute of Gombong-Central Java and Pharmacy Department of PKU Muhammadiyah Hospital, Yogyakarta, Indonesia; 2 Department of Health Behavior Environment Health and Social Medicine, Faculty of Medicine Nursing and Public Health, Universitas Gadjah Mada, Yogyakarta, Indonesia; 3 Division of Pharmacoepidemiology and Pharmacoeconomics, Department of Pharmacology and Therapeutics, Faculty of Medicine Nursing and Public Health, Universitas Gadjah Mada, Yogyakarta, Indonesia; 4 Division of Clinical Pharmacology and Medicine Policy, Department of Pharmacology and Therapeutics, Faculty of Medicine Nursing and Public Health, Universitas Gadjah Mada, Yogyakarta, Indonesia

**Keywords:** Rationing, Priority setting, INA-CBGs, Medicines in health insurance, Indonesia National Health Insurance

## Abstract

**Background:**

Universal Health Coverage (UHC) in Indonesia is planned to be fully implemented in 2019 through the National Health Insurance (NHI) launched in January 2014. However, limited financial resources cause health care providers (HCPs) to perform rationing in providing medicine services. The purpose of this study was to analyze rationing strategies performed by HCPs for potentially beneficial essential medicines due to financial constraints and other reasons in the Indonesian NHI Plan and evaluate its fairness.

**Methods:**

A qualitative study was conducted to find out the rationing performed by 24 HCPs in NHI medicine services at hospital setting. Research methods included semi-structured interviews with eight physicians, eight pharmacists and eight nurses, and observations of prescriptions undergoing dispensing process. Respondents were purposively selected, and interview results were analyzed thematically. The strategies for rationing were categorized using the matrix developed by Maybin and Klein (denial, selection, delay, deterrence, deflection, and dilution), while contradictions in fairness were evaluated using the four conditions of accountability for reasonableness (relevance, publicity, appeals, and enforcement).

**Results:**

The results showed that the most frequent rationing performed by physicians was dilution (to replace medicines with others which were perceived by physicians as less effective or less safe), denial (not to provide medicines not listed in the National Formulary and/or expensive medicine), and deterrence (to encourage patients to pay for medicine). Among pharmacists, the most frequently rationing performed was dilution (to reduce the amount of medicines), denial, and deterrence as performed by physicians. Almost no rationing strategy was performed by nurses. No formal procedure was available to guide the rationing. The rationale for rationing strategies, especially for non-clinical reasons, was often not communicated to patients, and there were few opportunities for patients to appeal the rationing strategies applied to them. There was no difference between the government and private hospitals in the rationing strategies adopted.

**Conclusions:**

Although rationing strategies were facilitating the implementation of National Formulary, they potentially raise problems related to the principles of medical ethics and distort a national health system’s ability to progress towards UHC. If performed in the more standardized decision-making process, rationing would be of great benefits to patients and the system. Guidance for more explicit, fair and transparent of rationing should be developed at the hospital level.

## Background

The Government of Indonesia has implemented a new National Social Security System *(Sistem Jaminan Sosial Nasional/SJSN)* during the last decade. The National Health Insurance Program (*Jaminan Kesehatan Nasional*/JKN) was launched on January 1, 2014, and the Government appoints the Social Insurance Administration Organization – Health (*Badan Penyelenggara Jaminan Sosial Kesehatan/BPJS-Kesehatan*) to organize the national insurance scheme with a target of the entire population by 2019.

The current report shows that around 198 million Indonesians have been enrolled. The ultimate goal of JKN is to ensure the access and equity of health care through all of the population including those in remote areas and islands. Many high-cost medical cares such as for cancer, hemodialysis, and cardiovascular surgeries were provided, which were before of luxury to the majority of people. This is the Government’s commitment to make UHC a national goal of health insurance system in Indonesia, as defined by the WHO as a condition that ensures that all people have access to promotive, preventive, curative, and rehabilitative services while ensuring that people do not experience financial difficulties when paying for these services [1].

BPJS-Kesehatan reported that cost of benefit in JKN had reached IDR 87,22 trillion in 2017 [2]. It is anticipated that the fiscal risk may reach IDR 16.31 trillion in 2018 if effective cost control is not applied [3]. To control the cost of health services in hospital setting, Indonesia Case Mix Based Groups (INA-CBGs) prospective payment system has been involved since the first implementation of the insurance program. The system is set from diagnosing and procedure grouping without count its kind and amount of health service provided. This case-based payment system aims to reduce the incentives for over-servicing and improves the quality and efficiency of health care [4].

Medicine cost is a part of health service which is included in the INA-CBGs payment system. To guide the medicine selection by health care providers, The National Formulary *(Formularium Nasional/Fornas)* has been established since the early implementation of JKN. Fornas is revised annually by the National Committee for Revision of National Formulary. Health providers have an obligation to follow the National Formulary when prescribing medicines for BPJS patients.

If the medicines prescribed by the providers are unavailable in stock or if the medicine cost exceeds the INA-CBG limit, rationing for medicine is sometimes practiced. In the other hand, studies showed that rationing was sometimes considered unfair due to its sub-optimum benefits to the patients [5]. A recent study reported that ad hoc or informal rationing could disadvantage the least well-off patients and distort the national health system’s ability to progress towards UHC [6].

Rationing has been classified in different ways. Among others, Klein’s classification appears to be the most frequently used in the literature [7], i.e., rationing by denial, rationing by selection, rationing by delay, rationing by deterrence, rationing by deflection, and rationing by dilution. This Klein’s classification was used to identify the rationing practiced by health providers under the National Health Service (NHS) in the United Kingdom (Table 1) [8]. The results flag the importance of evaluating rationing practices in Indonesia to refrain the rationing from the disadvantages for the patients.

**Table 1:** The matrix of Maybin and Klein’s NHS rationing strategies^a^ [8]

Form of Rationing	Description
Rationing by denial	This is the most headline-catching form of rationing. Specific forms of intervention are excluded from the NHS services on offer, on the grounds of lack of effectiveness, high cost or a combination of the two.
Rationing by selection	Service providers select those patients who are most likely to benefit from interventions or raise the threshold of eligibility for treatment.
Rationing by delay	The traditional form of rationing in the NHS, designed to control access to the system and match demand to supply by making patients wait.
Rationing by deterrence	If patients are not put off by queues, there are other ways of raising barriers to, and the costs of, entry into the health care system. Receptionists may be unhelpful, and information leaflets may be unavailable, or access may be difficult.
Rationing by deflection	All else failings, patients may be shunted off to another institution, agency or program. ‘Difficult cases’ may be referred to another hospital or specialist.
Rationing by dilution	Services or programs continue to be offered, but there are fewer nurses on the ward, doctors order fewer tests, the palatability of hospital food plunges, and the quality of care and treatment declines.

^a^cited with permission from Rudolf Klein

Klein emphasized that although it was important to distinguish between different forms of rationing, the result was the same: patients receive less than the best possible treatment [9]. In practice, rationing strategies often contain overlapping elements, which permit the application of more than one term to describe a specific situation. For example, delay and deterrence could be seen on a continuum with deflection and dilution serving as alternative options permitting an HCP to limit the provision of appropriate health care to such an extent that pertinent information was withheld, raising serious concerns about the fairness of rationing policies and their negative effect on patients’ autonomous rights, especially informed consent [10]. Besides patients’ autonomy as one of four principles of medical ethics, the other principles namely beneficence, non-maleficence and justice could be violated due to rationing by HCPs [5]. Daniels and Sabin [11] proposed the concept of “accountability for reasonableness” to better understand the underlying motivation in health care provision and institutional priority setting or rationing [12, 13]. This framework has been used nationally and internationally to evaluate the legitimacy and fairness of priority setting in many health care systems [14]. The ethical framework of Accountability for Reasonableness (A4R- reads as A for R) (Table 2) has been demonstrated to be useful in identifying ‘best medical practices’ and highlighting areas for improvement in decision-making [15], and has been applied as an excellent standard for assessing fairness of priority setting in drug formulary in hospital institutions [16].

**Table 2:** The four conditions of accountability for reasonableness (A4R)

Conditions	Description
Relevance	Rationales for limit-setting decisions must rest on reasons (information and values) that fair-minded parties (managers, clinicians, patients, and affected others) can agree are relevant to meeting health care needs under resources constraints in the priority setting context
Publicity	Limit-setting decisions and their rationales must be publicly accessible
Appeals	There is a mechanism for challenge and dispute resolution regarding limit-setting decisions, including the opportunity for revising decisions in light of further evidence or arguments
Enforcement	There is either voluntary or public regulation of the process to ensure that the first three conditions are met.

Source: adapted from Daniels and Sabin [11]

The purpose of this study was to analyze rationing strategies have taken and decisions made by physicians, pharmacists, and nurses to withhold potentially beneficial medicines under financial restrictions in JKN. This study also evaluated the decision-making process in regards to its fairness and legitimacy, by using the A4R ethical framework to assess “accountability for reasonableness.” After describing the rationing process using the perspectives of those health care providers and evaluating it with a relevant framework, appropriate recommendations were generated regarding areas for improvement of best practices in medicine services in the Indonesian National Health Insurance System.

## Methods

This study is a multi-case study with a qualitative approach, which consists of two parts of the study conducted simultaneously. The first part was an exploratory study to identify rationing strategies practiced by Health Care Providers (HCPs) namely physicians, pharmacists, and nurses at 4 public and 4 private hospitals in a province in Indonesia. The second part was an observation to prescriptions collected during the first part of the study at the same hospitals, to obtain information regarding changes in the medicine dispensing. Recruitment of the HCPs to the study was based on their exposure to BPJS patients.

Semi-structured interviews were conducted between September 2016 and October 2017 to a physician, a pharmacist, and a nurse of each hospital to gain perspectives of main actors of health care and the reasons for rationing strategies from the different profession in the different hospital. Altogether, 24 selected key respondents were involved, consisted of eight physicians, eight pharmacists, and eight nurses.

The key respondents were selected purposively based on their hands-on knowledge and duty, i.e., physicians who serve the biggest number of BPJS patients, clinical pharmacists, and nurses working at BPJS Wards. Triangulation of information provided by key respondents was undertaken to validate the findings. Twenty-four interviews were conducted, and saturation among professions and hospitals had been attained.

The interview guide was developed based on the rationing classification as described by Maybin and Klein’ matrix combined with Daniel and Sabin’s A4R framework. The interviews were conducted at convenient locations determined by participants at their workplace. Topics of the interview included general attitudes concerning financial limitations, views, experiences, and reasons for rationing for medicines to BPJS patients. The interview guide was checked for clarity and understanding involving physician and pharmacy colleagues at a hospital not assigned as the study site. Each interview was last in half to one hour.

In the second part of the study, prescriptions received by 30 randomly selected patients during the process of dispensing in parallel with the interviews were observed in each hospital pharmacy, resulting in a total of 241 prescription sheets of 240 patients. Data collection included the name and the amount of medicines when prescribed, and changes made in the name and amount of medicines when dispensed, Observation on medicines which were not dispensed were collected from the prescription copies given to the patients. No interview was conducted to those patients.

Thematic content analysis was applied to the results of semi-structured interviews. Using a modified open coding technique, the data were examined, and elements of rationing strategies and process were identified and described. Initial codes were then built into categories, and finally, the categories were organized using the Maybin and Klein’s rationing matrix [8] and the four conditions of A4R [11]. The prescriptions were analyzed using descriptive analysis. Prescriptions by physicians were compared to the medicines dispensed by pharmacists, and the differences reflected the results of rationing. Triangulation was applied for different key respondents (physicians, pharmacists, nurses) and data sources (interviews and prescription observation).

### Research ethics

The protocol of the study has been approved by the Medical and Health Research Ethics Committee of Faculty of Medicine of Universitas Gadjah Mada – Dr. Sardjito General Hospital, Yogyakarta with reference number KE/FK/1105/EC/2016. All respondents signed written informed consents. The identity of respondents was kept confidential at all stages of the research and publications.

## Results

Results are presented according to the rationing strategies performed by 24 HCPs: eight physicians, eight pharmacists and eight nurses based on their recognition and the evaluation of the decision-making process using the four conditions of accountability for reasonableness: relevance, publicity, appeals, and enforcement. The average years of respondents’ experience in the current hospitals were 10.71 (range 2–28). Characteristics of respondents are shown in Table 3. The results of prescription observations are presented at the end of this section.

**Table 3:** Characteristics of respondents

Characteristics	Number of respondents
Profession	
Physician (internist, neurologist, ophthalmologist)	8
Pharmacist (clinical pharmacist)	8
Nurse (BPJS wards)	8
Sex	
Female	15
Male	9
Age (years)	
Less than 40	12
40 or older	12
Years of practice in the current hospital (years)	
Less than 10	14
10 or longer	10

Generally, the findings showed that rationing is likely to be done in situations where patients need potentially beneficial medicines, but are not included in the JKN benefit scheme as stated in the National Formulary hereinafter referred to as non-Fornas medicines and/or the medicines which are expensive. Rationing strategies identified as done by health care providers are presented in Table 4.

**Table 4:** Rationing strategies by healthcare providers in four public and four private hospitals in a province in Indonesia using Maybin and Klein’s six categories

Rationing strategy		GH1	GH2	GH3	GH4	PH1	PH2	PH3	PH4
Not giving medicines (denial)	D	─	√	√	─	√	√	─	√
	P	√	√	─	√	√	√	√	√
	N	─	─	─	─	─	─	─	─
Select patients who will receive medicines (selection)	D	√	─	─	─	─	─	─	─
	P	√	─	─	─	─	√	─	√
	N	─	─	─	─	─	─	─	─
Encourage patients to purchase uncovered medicines (deterrence)	D	√	√	√	─	─	√	─	√
	P	√	─	─	√	─	√	√	√
	N	─	─	─	─	─	√	─	√
Refer patients to other health care facilities (deflection)	D	√	─	─	─	─	─	─	─
	P	─	─	─	─	─	─	─	─
	N	─	─	─	─	─	─	─	─
Replace with other medicines which less effective or less safe/reduce the amount of medicines (dilution)	D	─	√	√	√	─	√	√	√
	P	√	√	√	√	√	√	√	√
	N	─	─	─	─	─	─	─	─
Applied waiting list to obtain medicines (delay)	D	─	─	─	─	─	─	─	─
	P	─	─	─	─	─	─	─	─
	N	─	─	─	─	─	─	─	─

*D* Doctor/Physician, *P* Pharmacist, *N* Nurse, *GH* Government Hospital PH: Private Hospital

√: rationing was practiced; ─: rationing was nor practiced

### Rationing by physicians

From the interviews, it turned out that all of the physician respondents have performed many different strategies of rationing when serving BPJS patients. Three of four physician respondents from the public hospitals indicated they did rationing rarely due to the leniency by their hospitals, while the physician respondents from private hospitals often did rationing because they were worried about hospital financial losses and possible deficits in inventory.

Of the six strategies, denial was performed mostly by the physician respondents under situations when the medicines needed by the patient were too expensive and caused the patients’ medical costs greater than the claim using tariff of INA CBGs. The physician respondents complained that tariff of INA CBGs are insufficient for most of the diseases treated in their hospital which made them emphasize the cost of medicines and the tariff of INA CBGs as main criteria for denial strategy. None of the physicians applied cost-effectiveness analysis when making rationing decisions. The physician respondents admitted that reasons related to financial limitation were not easy to communicate with the patients, so all of them did not tell the patients this rationing strategy and the considerations. An example of the situation when a physician respondent applied denial strategy was when he had to treat a BPJS patient diagnosed with Guillain Barre Syndrome (GBS), and the patient needed intravenous immunoglobulin (IVIG) preparation. The following quote illustrates the physician’s response:

*“GBS. In the guideline (of GBS) the IVIG (is the drug of choice). The price of IVIG per vial is -the cheapest one, 2.5 grams- about 2 million (IDR). So, if you use it, it almost (costs) 100 (million IDR) or must be more. I once used 5 grams (of original IVIG) for a (non JKN) patient, and it cost 220 million (IDR). The JKN claims (tariff set in INA CBGs) was only about 5 (million IDR). If we used generic (IVIG, the costs would be) 100 million IDR, while only 5 (million IDR) was collectible, so 95 million were also borne by the hospital.”* A physician from a private hospital

Selection strategy was also performed by physicians. In some cases, physicians continued to prescribe expensive or non-Fornas medicines to particular patients. Upon the decision, some of those patients were up-graded to the higher class of care to obtain higher tariff of INA-CBGs, and some others were offered cost-sharing of medicine expenses. The difference of class of care and the tariff of INA CBGs that follow it were the primary reasons for this rationing strategy. These considerations were not published to the patients due to the contrary to the regulation that denotes the difference tariff of INA CBGs between class ward based on the facilities offered. Piracetam, citicoline, and mecobalamine for neurology cases were examples of this rationing strategy.

Another rationing strategy was deterrence. Physician respondents encouraged patients to purchase medicines not covered by BPJS when they knew the patients personally (friends, relatives, etc.), or when the medicines were affordable to the patients, or when they knew that patients had used the medicines for a long time and felt comfortable with these medicines and adhered to the treatment. Patients’ willingness to pay was the main consideration, in addition to reasons related to individual patient clinical conditions, hospital priority setting policy, and improving patients’ adherence. Ketoacid prescription for Chronic Kidney Disease patients was an example of this case. The clinical reasons for this rationing strategy were communicated to the patients by some of the physician respondents, and none of the patients challenged this rationing strategy.

In other cases, the physician respondents stated that they followed the Fornas restrictions for the rationing strategy. However, they felt worried as they perceived that the Fornas medicines they prescribed had lower efficacy and safety profiles. One of the physicians expressed their regret in prescribing less safe anti-epileptic medicines to pregnant women for the reason that the ‘safer’ medicine was not available at secondary hospitals and only available at the tertiary hospital. Although the physician’s opinion might not be absolutely correct, this feeling of ‘lowering the quality of treatment’ is classified as dilution strategy. The following quote by the physician respondent illustrates this case:

*“There was a (guilty) sense because I know this patient (a pregnant woman who suffers epilepsy) should be given a better medicine, there is something better. Even though it (the epilepsy medicine was given to the patient) does not threaten life, but (the patient) should be given a newer, better medicine and existed in Fornas. Why can it not be given to this patient who was treated in secondary referral hospital? The restriction of Fornas to this case is inappropriate..”* A physician from a public hospital

Of similar situation described above, a physician respondent claimed to refer patients to the tertiary hospital to obtain medicines which were only available at a higher level. This strategy was classified as deflection. The availability of the medicine in his hospital and the restriction on Fornas were the reasons for deflection. These constraints were informed to the patients, and the patients were mostly able to accept and did not object.

### Rationing by pharmacists

The interviews among pharmacist respondents revealed that they used some different rationing strategies. The most prominent strategy was rationing by dilution. Non-Fornas and/or expensive medicines were kept dispensed to patients as written in the prescriptions but in less amount, especially to outpatients. Symptomatic medicines were often given for three to seven days of treatment instead of longer duration as prescribed. Dispensing fewer amounts of antibiotics, vitamins and statins were other examples of this strategy. In some cases, pharmacists asked physicians to change expensive medicines to cheaper brands or to other active compounds which might be less effective or less safe to patients.

Denial was the second most common rationing strategy conducted by pharmacists. Some pharmacists decided not to deliver certain prescribed medicines because they thought the price was high and those items were non-essential, such as vitamins or supplements. In some cases, those medicines were delivered to patients who occupied wards with higher INA CBGs rates or wards that allowed copayment or cost-sharing for the cost deviation. This strategy was classified as selection. Rationing by selection was also performed to patients whose medical costs have reached the maximum limit for reimbursement or exceeded the INA CBGs tariff.

Some pharmacists admitted that the patients received the most rationing were those who occupied the lowest ward Class 3 or patients whose premiums paid by the Government or contribution beneficiaries (*Penerima Bantuan Iuran*/*PBI*). Denial and dilution strategies overlapped with the other strategy, namely deterrence. Medicines that were not given by the pharmacist or remaining medicines prescribed by the physicians were informed to the patients who were encouraged to purchase them outside the hospital. Just like physicians, pharmacists in private hospitals were more active in conducting rationing because they worried about hospital financial losses.

Pharmacists were instead more interested in the price of medicines and the sufficiency of tariff set in INA-CBGs prospective payment than cost-effectiveness analysis in decision-making strategies regarding rationing medicines in JKN. They used rationing strategies with the implicit reason for the budgetary restriction. Workload and inadequate pharmacoeconomic knowledge were the impediments for pharmacists to conduct cost-effectiveness analysis.

Most of the pharmacist respondents complained about the insufficient tariff for INA CBGs for most of the diseases in their hospital, and this caused them to feel that they were the health care providers most burdened to control the cost of therapy in BPJS patients. In addition to the financial consideration as described above, pharmacists also referred to the existing Fornas restriction to use medicines based on diagnosis or results of laboratory examinations. The following quotes illustrate some of the reasons by pharmacists in doing rationing for medicines to BPJS patients:

*“So, sometimes, if we wanted to give (the medicines) which do not exist (in Fornas), it was a dilemma. We had to count (the cost). The other case was thalassemia, the medicine is expensive, while the quota (tariff set in INA CBGs) is only about 1 million (IDR). Well, if we wanted to give the medicine to patient who stayed in the hospital, we asked the (nurse in) ward what costs have been used by patients for other than medicines, and the (amount of) medicines for patient discharged will be calculated and adjusted according to the rest (of the money in INA CBGs rate)”.* A pharmacist from a public hospital

*“They will be provided with a prescription to buy the medicines by him/herself. The prescription was not recorded in the patient history transactions. So, it’s like buying vitamins. Yes, it could have happened, when the patient wants to buy it by him/herself. Usually, (the patients) have been motivated by the physician. Actually, it is not permitted by JKN system, but what else I can do”.* A pharmacist from a private hospital

Some pharmacists tried to explain to patients the reasons for the rationing they had been doing, especially to outpatients. Although the reduction in the amount of medicines or changing medicines was under the hospital management decision, the absence of official publication to the patients made the pharmacists at the risk of complained by patients. Nearly all hospitals allowed pharmacists to communicate medication changes with physicians, only one hospital issued an official decision regarding the reduction in the amount of medicines given to JKN patients, and only one pharmacist discussed with the prescribing physicians in reducing the amount of medicines delivered.

A few patients and physicians appealed about the rationing done by the pharmacists. When it happened, the pharmacists revised their rationing decisions to avoid conflicts and to avoid prolonged service time. Almost all hospitals did not have clear procedures to regulate the reduction in the amount of medicines, and none of the hospitals involved in this study had any regulation regarding drug fees paid from patients because JKN regulations discourage patients from paying out of pocket for the medicines they received.

### Rationing by nurses

Unlike physicians and pharmacists, there was almost no rationing practiced by nurses, except a nurse who was a member of the team looking after expensive or non-Fornas prescriptions for BPJS patients. Only two nurses from private hospitals stated having advised patients to purchase medicines which were not included in the JKN benefit scheme. This strategy was classified as deterrence.

Nurses in government hospitals claimed there were a few situations that required rationing because their respective hospitals provided rooms for flexibility in medicine prescription. However, all nurses recognized their role as the party responsible for providing accurate information to patients about rationing decided by management, physicians or pharmacists. Most nurses claimed one of their duties was administering medicines to patients, regardless of patient care costs or medicine prices. But, it was not uncommon for some of them to convey information to physicians if the medicines could not be given to patients because the price was too high or not covered by BPJS benefit scheme or would exceed the INA CBGs tariffs.

### The fairness of rationing by health care providers

The rationing decisions discussed in this study were highly context-dependent decisions involving clinical and non-clinical reasons. Non-clinical reasons such as the cost of medicine, tariff set in INA CBGs, restrictions in National Formulary, and drug availability were the major considerations that emerged from this study for most of the HCPs in explaining rationing strategies, and pharmacists paid more attention to these reasons. They had to remind the physicians or report the case to management (board of directors), or they made their rationing if the medicines prescribed by physicians were expensive, not listed or did not fulfill restrictions in National Formulary or the cost of therapy extended from tariff set in INA CBGs.

Individual clinical conditions of patients were the other criteria by physicians. Local (hospital) policy of priority setting was considered by a few HCPs. Only one physician and one pharmacist from a private hospital stated that drug efficacy and side effects were considered. Conformity with international or national guidelines including clinical pathways which were developed by hospitals was used by some of the HCPs in rationing decisions.

Unfortunately, clinical pathways of many diseases, especially complicated ones, have not been established by hospitals. Some existing clinical guidelines did not apply to JKN because they do not match the INA CBGs rates. However, hospitals have not yet developed cost-conscious clinical guidelines. External factors such as the ward where the patient stayed were also a consideration due to the different regulations between the wards. The patients’ demands and their willingness to pay for medicines was also another reason for rationing by all the HCPs.

Reasons related to the context in which rationing occurs were not well communicated by physicians to patients because they claimed not to have enough time to explain this issue. To patients, especially civil servants who did not get medicines or got ones that were different from those obtained from previous health insurance, physicians and pharmacists attempted to convince them that the medicines given were as good as those previously prescribed. Nurses were the providers who most communicated the reasons for rationing conducted by physicians and pharmacists. Many tended to frame the reasons for the patients as purely clinical when they were not. Reasons related to financial limitations that are too burdensome and they lacked the skills to articulate this kind of issue.

An appeal mechanism was not formally provided to parties who objected to rationing conducted by physicians or pharmacists. Challenges were mainly raised by patients who pay their premiums or noncontributing beneficiaries in government and private hospitals. Pharmacists were likely the target of patients’ appeals compared to physicians, and sometimes they were forced to revise the rationing done by other HCPs to avoid conflict and prolonged medicine service time.

No hospital officially regulated the rationing done by HCPs. Hospital regulations only specified additional non-Fornas medicines that can be given to BPJS patients. In some cases, pharmacy and therapeutic committees or the board of directors encouraged providers to make rationing for which the implementation was entirely handed over to each provider. Almost all hospitals allowed pharmacists to replace expensive medicines with cheaper ones by making a generic substitution, but only one local government hospital officially stipulated restrictions on the actual amount of drugs delivered to patients. One government hospital had a Director’s decree stating that high-cost medicines’ administration required approval from the Board of Directors and Medical Committee.

### Changes in medicines dispensed due to rationing

Observations were made on 241 prescriptions received by 240 patients. The total numbers of medicines prescribed by physicians were 1561. From the observation, it turned out that only small percentages of medicines were changed. Medicines substitution was performed in 5.8% of the total medicines prescribed, and only 5.1% of medicines prescribed were received by patients at a smaller amount. Two point six (2.6) percent of the medicines were not dispensed; 2 % of these medicines were proposed for purchase by patients. Details of the changes are presented in Table 5. It was also observed that all medicines prescribed for chronic diseases were dispensed in full amount. For other purposes of the treatment, medicines were frequently dispensed for 3–7 days of use regardless of the duration of treatment prescribed by the physicians.

**Table 5:** Changes in medicines dispensed due to rationing

Rationing results	GH1	GH2	GH3	GH4	PH5	PH6	PH7	PH8	Total (%)
# of total drug items which were prescribed by physicians	268	175	246	207	172	181	136	176	1561
# items which were substituted by the pharmacist	22	11	2	25	20	6	1	4	91 (5.8)
# of items which were given less than the amount prescribed	14	6	0	30	2	19	2	7	134 (5.1)
# of items which were not dispensed	1	6	2	2	5	4	9	12	41 (2.6)
# of items that were advised for the patient’s purchase	1	6	0	2	5	11	1	5	31 (2)

*GH* Government Hospital, *PH* Private Hospital

Based on the observations, the second most common rationing after dilution was seemed to be denial. Total of 41 items of medicines was not given to patients due to several reasons, i.e., they were not listed in Fornas, medicines were not in stock, or patients still kept the medicines from the previous consultation. Of the 41 medicines not dispensed, patients were advised to buy 31 medicines. The later was categorized as deterrence strategy.

## Discussion

Indonesia National Health Insurance System has been established for 5 years, but studies and information related to medicines services are limited. The previous studies mostly conducted to measure implementation of Fornas quantitatively, cost of medicines in the therapy of BPJS patient compared to tariff INA CBGs, and out of pocket incident by BPJS patients. On the other hand, complaints related to medicine service still arise from HCPs and BPJS patients. The lack of information related to medicine service like medicines was not given to the BPJS patients which is referred to rationing made some people judged that BPJS patients get a low quality of medicines services. This study provides clearer and wider information about the process behind the medicine services to BPJS patients in hospitals at financial limitation, including the process of not giving some medicines for several reasons. The qualitative study offers advantages by uncovering the phenomenon that had not been revealed before [17].

Using Maybin and Klein’s matrix of rationing strategies [8], this study found that most of the HCPs have performed various rationing strategies. Denial was performed by physicians for High-Cost Medicines (HCMs) in the situations when the medical cost of the patient exceeded the INA CBG tariffs. This strategy potentially violates the beneficence principle of medical ethics that HCP has an obligation to help people in poor health [5]. Pharmacists performed denial strategy in the situation when the medicine price is high, and the item was non-essential such as vitamins and supplements. Pharmacists were aware of the implementation of Fornas and maintained the rational use of medicines. None of the HCPs in this study used cost-effectiveness analysis as a tool for making rationing decisions due to lack of capacity and not having enough time to do the analysis.

Reviews by Vuorenkoski et al. [18] showed the same result that formal pharmacoeconomic analyses were rather seldom used, partly due to quality issue in the analysis and lack of knowledge on health economic evaluation. A survey of rationing on 1032 physicians showed that most (67%) of the physicians supported cost-containments, while over half (54%) objected to applying cost-effectiveness data in clinical decisions [19], and until recently economic approaches like cost-effectiveness were limited in priority setting agendas for most institutions [13]. Difficulties expressed by physicians in using high-cost medicines (HCM) might be improved by involving more experts and group practice with a more transparent process. In public hospitals in Australia, HCMs are defined as those costing more than AU$5000 ((€3000/U$3800) and using these medicines is regulated by High-Cost Drug Sub-Committee (HCD-SC) [20].

Selection strategy was performed by HCPs in the situation when HCM is needed but exceeding the INA CBGs tariff, by upgrading patients to the higher class. Although not explicitly stated by HCPs, patients who occupied in a higher class or higher INA CBGs rates or wards have a greater chance to obtain HCMs or Non-Fornas medicines. The action of upgrading selective patients might violate the principle of justice, one of the four principles of medical ethics [5]. Both physicians and pharmacists should have made a fair process in this selection strategy to prevent a lawsuit from other parties who did not enjoy the privilege.

The next rationing strategy was deterrence. Access to expensive and/or non-Fornas medicine was complicated by requesting patients to purchase out of pocket the medicines they needed. Implicit criteria like the patient-physician relationship, patient comfort and ease of use of medicines were important criteria in deterrence strategy performed by physicians. These findings correspond with Gallego et al. [20] who described there was a considerable difference in emotional responses of physicians when dealing with a familiar patient who may benefit from treatment compared with an unfamiliar group of people. The pharmacists tended to do deterrence strategy more due to medicine price consideration, Fornas restriction, and cost of therapy. This result is consistent with previous studies which showed pharmacists more often made rationing decisions based on concerns about acquisition cost and budgetary overspending [21].

Rationing by deterrence has the potential to cause dissatisfaction and make the patients suffer financial hardship, and as a result, the medications needed are not obtained. A systematic review and meta-analysis found copayments for prescriptions medicines among publicly insured patients increased odds of non-adherence by 11% [22]. Another study found a significant reduction in the use of essential drugs and related higher rates of serious adverse events and emergency department visits after there were increases in cost-sharing for medicines prescribed to marginalized populations including the elderly and recipients of welfare programs [23]. The principle of medical ethics namely justice has been potentially violated by HCPs in this strategy. The principle of distributive justice requires all people to equally receive a reasonable level of medical services based on medical need without regards to the ability to pay [5]. This rationing strategy is potentially in contrary with JKN regulations which stipulate that patients insured under the JKN should have access to cost-free medicines within reasonable demands [24].

Deflection is the rationing strategy that is possible to perform due to in line with the Fornas restriction and the referral system. However, this strategy is uneasy to apply due to bed availability of the referral hospital, or due to patients’ objection. In this situation, patients’ autonomy to choose a hospital or physician where he/she feels comfortable, one of the principles of medical ethics should be considered.

This study found that the most common strategy of rationing done by HCPs was dilution, but the presumption of some HCPs that the medicines in Fornas are less effective or less safe need to be straightened out. The existing restrictions of medicines listed in Fornas were the main consideration for physicians and pharmacists, but the cost of medicine and tariffs set in INA CBGs were major reasons for pharmacists when engaging in dilution of essential medicines. Dilution has potentially violated the non-maleficence principle of the medical ethics that HCPs should do no harm [5] if the reduction of the amount of medicines dispensed is in contrary with treatment guideline. The hospital’s local policy often contributed to the basis for dilution decisions by pharmacists, but only one hospital issued an official decision on reducing the amount of drugs given to patients. In the view of the successful implementation of Fornas, rationing strategies that refer to Fornas restriction is fundamental.

Availability of medicines in stock was another reason for many rationing strategies by HCPs. Some physicians admitted that sometimes they have to do rationing due to drug shortages and pharmacists made decisions in rationing for selected patients if the essential medicines were limited. Some researchers recommended evidence-based practice for optimal allocation when prioritizing patients during a shortage of medicine. Iyengar et al. [25] recommend the use of A4R ethical framework when dealing with shortages.

The clinical condition of patients was another consideration. However, there was no consensus or guidance at hospital level on what clinical conditions that physician should take a decision on rationing. A systematic review by Strech et al. categorized the above situation as implicit rationing strategy, and this was the most widely practiced [26]. Some hospitals made rationing decision based on explicit rule-based decisions such as hospital formulary, severity of the disease, treatment effectiveness, or clinical guidelines for cost-effectiveness.

Research shows that physicians’ rationing behavior tends to be extremely variable, largely caused by context-related factors, and involving primarily implicit rationing strategies [20]. This study found that rationing by physicians was influenced by various social and psychological factors, while rationing by pharmacists was based on explicit institutional regulation or formulary restrictions.

Based on the principles of A4R ([11], the rationing must rest on reasons (information and values) that agreed by fair-minded parties (managers, clinicians, patients and affected others) and relevant to meeting health care needs under resource constraints. Erntoft [21] recommends the development of clinical guidelines based on cost-conscious as a tool to make a better rationing. The clinical guidelines were developed through a fair and equitable process with notice tariffs set in INA CBGs and patients’ ward occupied are recommended to the one of the guidance for HCPs in making rationing strategies.

The reasons for rationing were not always communicated to all stakeholders especially to patients and were not usually accessible to the public. Non-clinical reasons were not easy to communicate and potentially sued by the public. HCPs especially nurses usually justified the rationing on clinical reasons when in fact they were not. Not all rationing conducted by pharmacists were communicated to the physicians, but they were usually communicated to the patients. This pattern has the potential to decrease the quality of medicine services to BPJS patients, to cause the patient’s dissatisfaction and threaten the trust in the Universal Health Coverage program.

Presently, no appeal mechanism is officially offered to patients against the rationing they received. Only a very few patients mainly non-PBI challenged the rationing done by pharmacists, and it made the pharmacists revised their decisions. No standard operating procedure exists to ensure that the afore-mentioned criteria can be accepted by managers, clinicians, and patients. These criteria for rationing were not communicated to patients, and therefore patients had no chance to challenge if they disagreed. These conditions showed that the rationing for medicines done by HCPs lacked in transparency and accountability. This needs immediate improvement as recently described in previous research findings [27, 28].

This lack of transparency potentially hampered efforts to achieve sustainable UHC as stated by Wagner et al. [29] that transparency in setting medicine coverage under resource constraints will be a lever for sustainable UHC. The results of this study revealed that there was no difference in rationing strategies between HCPs in public and private hospitals. This result is slightly different from the research conducted in Ghana which shows a difference between private and public providers in the quality of pharmaceutical services under Ghana’s National Health Insurance Schemes [30]. This problem of rationing by HCPs needs some improvements in the enforcement of the system at national, hospital, and bed site levels. At the national level, Indonesia Health Insurance’s should discharge the financial deficit to set the cost tariff of INA CBGs based on more reasonable prices. National standard therapeutic guidelines, especially for high-cost diseases, should be based on cost-conscious, fair and equitable processes and then be used as the basis to set the INA CBGs. The existing mechanisms for top up for high-cost medicines (for example, streptokinase, deferiprone, deferoxamine, deferasirox, and human albumin) should be maintained, and expansion of the drug list should be conducted based on cost-effectiveness analysis.

Similar to the national level, hospital decision makers should favor drug efficacy and safety concerns above cost considerations. In a shortage of medicines, it is necessary to prioritize patients based on evidence-based practice. Health technology assessment (HTA) which is already introduced and included as one of the national hospital accreditation standards should be implemented in decision making for hospital medicine lists and hospital clinical pathways, mainly for high-cost and non-Fornas medicines. Although hospital-based HTA still faces barriers [31], the availability of solid processes and tools to guide decisions on innovative and new medicines or high-cost medicines are necessary [32].

### Limitations

The limitation of this study is generalizability. This study was conducted in eight secondary referral hospitals in a province in Indonesia which might not be the same with other types of hospitals with different problems of diseases. HCPs in hospitals serving more patients with more complex diseases may have different issues than those who participated in this research. However, as rationing for medicines is something that all HCPs who work in hospitals must face, it is likely that other HCPs in other hospitals may see a reflection of themselves in the findings.

The lessons from this study can be used by other hospitals to improve their rationing decision to achieve best practices toward the comprehensive target of the Indonesia Universal Health Coverage in 2019. Still, the result of this study could not be representative for the public health centers where the medicines are supplied by district governments or hospitals in remote and geographically difficult areas where problems of stock-outs may be more frequent. Hospitals implementing other prospective payment system need to translate the lessons learned into their context. Further study with wider hospital population needs to be performed.

## Conclusions

Prospective payment system INA-CBGs tariff in Indonesia National Health Insurance sometimes makes HCPs have to perform rationing in prescribing and dispensing medicines to patients. Dilution, denial, and deterrence were the most rationing strategy performed by physicians and pharmacists, while almost no rationing strategy was performed by nurses. Although the rationing strategies that refer to Fornas restriction facilitating the implementation of National Formulary, rationing also potentially raise problems related to the principles of medical ethics either to beneficence, non-maleficence, autonomy, or justice principle. This condition can cause inconsistency and disadvantage socio-economically vulnerable individuals/households and distort a national health system’s ability to progress towards UHC. However, if performed in the more standardized decision-making process rationing would be of great benefits to patients and the system. The availability of a guidance for more explicit, fair and transparent of rationing processes and tools to guide decisions on innovative and new medicines or high-cost medicines are necessary. Supports of key stakeholders including health professional associations are strongly needed.
